# Case Reports May Be Declared Dead by the *Journal of Clinical Microbiology*, but They Are Alive and Well in *JMM Case Reports*

**DOI:** 10.1128/JCM.02870-15

**Published:** 2016-01-28

**Authors:** Johannes G. Kusters, Robert H. Hall

**Affiliations:** a Department of Medical Microbiology, University Medical Center Utrecht, Utrecht, The Netherlands; b The Microbiology Society, London, United Kingdom

## LETTER

We were disappointed to read the *Journal of Clinical Microbiology* (JCM) editor in chief's recent editorial “An Obituary for the Case Report in *Journal of Clinical Microbiology*” (1).

Despite the steady increase in the number of case reports published, the editorial board reportedly decided that case reports seldom add truly significant information to the medical literature, often lack true novelty, and are infrequently cited. The United Kingdom-based Microbiology Society reached a different conclusion and in January 2014 launched a sister publication to the *Journal of Medical Microbiology*, namely, *JMM Case Reports* (http://jmmcr.microbiologyresearch.org/content/journal/jmmcr). Historically, case reports have confirmed the etiologies of infectious diseases, alerted physicians and veterinarians to new pathogens and new presentations, communicated procedures and outcomes in patient care, and established archetype organisms for detailed research. We contend that case reports can still provide a valuable link between the patient and those working at the bench, bedside, or in the field.

Case reports provide an ideal format to communicate initial observations, interventions, and experience with novel pathogens and atypical patients. Case reports can alert clinicians to emerging infections and clinically relevant evolutionary changes in pathogens, such as drug resistance and hyper-virulence. The case report format also confers the specific, rapid, and precise description of events that are eventually aggregated into surveillance and epidemiological studies. Case reports have provided the clinical seeds from which grow the large-scale multicenter studies that provide the rational evidence upon which modern best clinical practice relies. Case reports give the profession the opportunity to research contemporary pathology and clinically relevant isolates, in place of ancient cases and archival type species. Because contributions to *JMM Case Reports* come from all over the world, the case report also provides an intriguing insight into contemporary infectious disease management in diverse, often challenging, settings.

Recent regulatory changes can make large clinical studies prohibitively expensive or ethically unjustifiable. In the latter case, it is often necessary to revert to evidence from animal or other experimental models, even though actual evidence on the effectiveness of alternative therapies, vaccines, or diagnostic methods may have been collected from cases in the natural host. Disseminating such information requires a platform to publish peer-reviewed case reports.

Our surveys have also revealed a relatively low level of citations; however, hits and downloads from the journal website are encouraging. We understand that although case report information is frequently used in daily clinical practice, this use is not recorded as a formal citation in an indexed academic journal, and although impact factors and citations exert an ever-increasing and indeed compelling influence on research, funding, and publications, it remains unproven whether these metrics predict the value of research to improving health outcomes. The poor impact scores achieved by case reports may reflect the irrelevance and overinterpretation of the metric.

So, although we are sad to hear that JCM has carried their case reports to the grave, we encourage scientists and clinicians to browse *JMM Case Reports*, and we invite submissions in medical and veterinary fields. Time will tell if the broad community supports a publication dedicated to case reports.
